# Was Robert Houston, of Glasgow, the First Ovariotomist?

**Published:** 1897-06

**Authors:** Lawson Tait

**Affiliations:** New York and Albany; L. R. C. P. Edin., etc., Birmingham, Eng. 7 The Crescent


### BUFFALO MEDICAL JOURNAL.

Vol. XXXVI. JUNE, 1897. No. 11.

### Original Communications.

### WAS ROBERT HOUSTON, OF GLASGOW, THE FIRST OVARIOTOMIST?

By LAWSON TAIT, M. D. New York and Albany; L. R. C. P. Edin., etc., Birmingham, Eng.

DR. GREIG SMITH has favored me with a reprint copy of an article by him in the *Practitioner* for March, in which he answers this question in the negative.

For my own part I regard all such matters as mere dialectic curiosities; and so far as concerns any possible claim of such a kind made for myself I should not care one pin what the answer might be. Others, however, attach importance to them, and Mrs. ———, the granddaughter of Ephraim McDowell, has gone so far as to class me in the list of those who have "attacked" her illustrious ancestor, because I have indicated a slight interest in the history of Robert Houston and have credited him with at least some importance in the history of this operation.

Dr. Greig Smith goes almost as far and has spent much literary skill and great industry in the preparation of a "library" paper. The misfortune of the paper is the absence of clinical acumen and logical accuracy, and it is this striking contrast in its constructive elements which induces me to reply to it, and to show, as I am sure I can do, that Robert Houston's case (1701) was an ovariotomy in every sense of the term and that (be it of interest and importance or not) it is the first case of that operation of which we have any record.

In all such efforts the demands of logic must be supplied by definitions agreed upon, and here, of course, Dr. Greig Smith and I may differ. If we should do so, our readers must judge between us. My first question for a definition, then, is, "What is an ovariotomy?" and I think the answer is quite easy: "A surgical operation by which it is thought to cure a tumor of an ovary." Of course, *one* ovary here includes *two*.

It will not do to say merely that it is a cutting operation, nor an *abdominal section*, because my old friend and colleague, the late Mr. Samuel Berry, did one ovariotomy in his long and honorable life—he removed an ovarian tumor through a rent in the vaginal flexure in the course of labor, tied the pedicle and cut off the tumor—a complete ovariotomy.

It will not do to say that the operation must be "intentional," for many ovariotomies completed were not begun with any intention of doing ovariotomy at all.

It will not do, as Dr. Greig Smith seems inclined to believe, that the whole of the tumor must be removed, for in many operations large areas of cyst walls have to be left; yet such may be perfectly successful and are, so far as necessity goes, complete.

It will not do to say a pedicle must be seen and tied, for in many of the cases where transplantation by twisting and gangrene division of the pedicle has occurred completely, the tumor is removed by enucleation and the pedicle is never seen. In some, indeed, the tumors have been found entirely loose in the abdomen.

It will not do, as Dr. Greig Smith does, to interlard the discussion with views on pathology as to whether or not Houston was right in them. For instance, he quotes "Showing that Houston believed, with most of his compeers, that 'ovarian dropsy' was simply a dropsy of the ovary." Singularly enough, that is my belief today, though I don't follow, as I cannot understand, what Dr. Greig Smith says further, "and not a tumor at all—and that he would as soon have suggested amputation for dropsy of the leg as excision for dropsy of the ovary." As I cannot understand, then, I may be forgiven if I say it is nonsense and quite beside the issue. We know very little of pathology; our advances alleged are generally confined to a change of nomenclature, with a very occasional and generally very unimportant extension of knowledge of minute structure.

Let us see how completely Dr. Greig Smith himself disregards this last difficulty and how completely I agree with him. He says: "Now, in considering Houston's case, we must be certain of the meaning of the words employed. The case undoubtedly records an operation of ovariotomy in the literal sense of the term, *incision* of an ovary or ovarian growth." Here he gives me my whole case, as I shall speedily show, only there is surely a misprint (or a very serious blunder in syntax on the part of Dr. Greig Smith). He should have said an incision *for*, not *of*. With this correction we are at one, for Houston is guilty of no such misstatement. It is clear he diagnosticated an ovarian dropsy and he opened the abdomen to cure it. He did not know what was going to happen as he went on—only the supernaturally wise amongst us know any better even now.

But Dr. Greig Smith goes on and this is the chief element of error in his argument. It is claimed for Houston that he did ovariotomy in the usual sense, but "for this last operation two things are essential: one, that the tumor should be delivered from the abdomen, and the other, that its connections with the body should be severed. It is unnecessary to go beyond these two essentials; and there is not even a suggestion that either was carried out." But they are not essentials, for it is not always either possible or necessary to deliver the tumor from the abdomen, as I have already shown; and in some cases there is no connection at all with the body, as I have also shown. Furthermore, the very details, given so accurately and effectively by Houston, shows that his was a case where Dr. Greig Smith's "essentials" were quite unnecessary and wholly impracticable. I find they are not essentials at all, in a very well-marked group of cases, of which Houston's was one, the first on record. Therefore it is that there is not a suggestion in Houston's record that either was carried out. Therefore it is that I made a mistake when, in 1883, I said "he must have seen and divided the pedicle." I was then as much in the dark as Dr. Greig Smith still is, and, like him, I believed in the "essentials;" but it is just because Houston so accurately describes what he did do and says nothing about that which he did not do that his story is absolutely deserving of credence and why he deserves the credit, be it worth what it may, of being the first to do an intentional, complete and successful ovariotomy.

Looking back on my thousand of ovariotomies, I see now that there are many distinct groups to be made; each of these groups includes numbers, great or small, which give characters different from the other groups, and these characters demand different views for their pathology and different methods for their treatment. Until our eyes are open it is astonishing how blind we are. Spencer Wells and others, myself included, went on removing healthy ovaries, along with simple broad ligament cysts, until it became quite apparent that they only required enucleation and were thereby cleanly brought out from their extraperitoneal bed without need, in most cases, of tying even one vessel. Removing the healthy ovary and tube was mere nonsense, and no one persisted more strenuously against other people doing it than Sir Spencer Wells, yet he went on doing it himself. The group of dermoid tumors is another far less common and having widely different conditions of pathology and treatment from any of the others.

Another group is one to which I venture to give, tentatively, the name of "digesting" tumors, for the phenomena of peritoneal digestion is certainly a leading feature in their history. There can be no doubt whatever of the accuracy of the extraordinary stories told of the disappearance of ovarian tumors. I have myself seen and diagnosticated ovarian tumors and have watched them disappear; and now that I have operated in quite a group of a special kind, and have caught a few of them in the actual process of disappearing, I am satisfied that they are all of one and the same kind, and that if we could only tell them in advance no operations would be necessary for most of them. But we cannot diagnosticate them, and they are too few in number to influence the general and best rule for ovarian tumors—remove them just as soon as the operation can be arranged after they have been recognised.

The group of which I now speak is not a large one, for whilst I am quite sure I have seen a dozen, I don't think I could dig out more than twenty in all, even if I gave myself up to the wearisome labor of seeking them in my enormous volumes of records, and I am not disposed to take up the useless task. I remember some of them very clearly, and I have complete records of three of them available; I have vivid recollections of some of the features of others and I have had several called to mind by the recollections of my former assistants and nurses. I can, therefore, give a fairly accurate picture of their peculiarities, but must leave it to others to confirm, extend or correct my observations, as may happen, when similar experiences arise to them. After reading what I have to say I think none of our younger ovariotomists will pass one of these cases without more careful investigation than any has yet received. The collection of the careful records of a few instances will prove of great interest.

Fortunately, I have already to hand two independent records which fit into mine exactly, the first of which is Houston's own; and the second an example recorded in the most imprejudiced and interesting manner by my colleague and former assistant, Mr. John

William Taylor, to whom I am obliged for full notes of the case. He describes the tumor as "a large multicystic tumor of the left ovary; contents semi-solid and gelatinous, which had already partially escaped into the peritoneum, causing chronic peritonitis." So far the details are precisely those of the group to which I refer, though the "chronic peritonitis," as I have noted the appearances, are more those of the gorged velvet of a mucous surface engaged in the full to its function, such as the stomach presents during the process of digestion. The further details supplied by Mr. Taylor do not help me, particularly the details of the pedicle, which was treated by a wire clamp. From Mr. Taylor's first and less complete description, I thought that the gelatinous contents went down to the base, but there it was more solid, and Mr. Taylor is of opinion that it would not have been possible to deal with it in any other way than the clamp, and I have no right to question his judgment.

As in other pathological groupings we have no hard and fast lines, so here we shall have complete and incomplete cases.

Fortunately, from my own experience, I can adduce a number of cases where the details are noted and where they are very clear in my own recollection and in those of my assistants.

The features to be noted are that the walls of the cyst are peculiarly thin and are perforated at the thinnest points, the contents of the cysts being freely extravasated into the peritoneum. In one or two of the cases I have seen more of the cyst contents in the cavity of the peritoneum than in the tumor. The peritoneal surface, especially the visceral surfaces, are intensely congested and often covered with small floculi, semi-transparent, but quite different from the purulent patches of lymph of chronic peritonitis, if carefully examined. My own belief is that the peritoneum is digesting and removing the exuded cyst eontents, and therefore presents a physiological appearance in so exaggerated a form as to appear pathological. The exuding contents of the cysts is always gelatinous or viscous, a most peculiar kind of stuff not like anything I know elsewhere. Houston's description of it is very good. It won't flow through any trocar and can be induced to get out of any sized wound only with the greatest difficulty. The hands are powerless to scoop it out, but it runs away rapidly with a stream of hot water, the only way of getting rid of it. As it dissolves in the warm water it becomes clear, as a rule, and shows clots as it flows in the stream, just as soap jelly does. When the abdomen is cleared, and the process is a long one, the cyst is found to be a miserable bundle of shreds with a vague pelvic attachment, whilst in the cleansing, pieces of cyst wall and secondary cyst masses are washed away. I have generally tied this diffused mass, cut it short and dropped it back, but in some six or eight cases, so far as my memory serves me, there was nothing left to tie. This was notably the fact in the first case of which I have clear recollection, a patient sent to me by Mr. Alfred Freer, of Stourbridge, and operated on by me in 1879. There nothing required to be tied. In another sent to me by ————, and operated by me in ———, the attachment of the tumors was a soft villous mass, on which two small bleeding points were secured and the rest left undisturbed.

The details in others vary considerably, but the main points are sufficient to indicate close similarity, and the differences in character from the ordinary fluid-containing ovarian tumors are such as to demand a distinct classification. Of course, in all these cases, I use a drainage-tube.

Mr. Christopher Martin, for several years my resident assistant, has favored me with his recollections of two cases. He writes:

> I find I operated on Miss S., on February 29, 1892, you assisting me. There was a ruptured cystoma of the right side; the abdomen was full of apple-jelly fluid, yellow and transparent, the tumor was almost sessile, the pedicle very friable; it tore through and had to be twice ligatured, and the abdomen was washed out and drained. I also remember the case at Swadlingcote, that of a middle-aged woman, on whom you operated. The abdomen was full of colloid stuff, which you washed out and drained. There was practically no pedicle to tie.

I had forgotten this last case until reminded of it by Mr. Martin's letter. She was 57 years of age, a patient of Dr. Hay Moir, of Swadlingcote, and I operated on her on June 15, 1892. The tumor was of the left ovary, but most of its contents had escaped into the abdomen, and I washed only a mass of débris, consisting of jelly and fragments of cysts. At the point of attachment in the pelvis was a mass of stringy fragments, which represented all there was for a pedicle, and nothing was tied.

The main features of the group are, the singular cyst contents, the rupture of the cyst walls, the extrusion of the contents, and the consequent activity of the peritoneal serous surface; the distinction of the cyst and the evidence of its loose detritus in the cavity of the peritoneum; and, generally, an exceedingly vague area of attachment of the pedicle and a marked absence of prominent bloodvessels.

It was only after a comparative leisure came to my life by the discontinuance of hospital work, that I began to think it worth while to stitch together casual notes and recollections, and the re-reading of Houston's vivid description fixed the group in my mind at once.

I said, in 1883, he must have tied the pedicle, but I know now that he didn't tie the pedicle because he doesn't say so, and because the pedicle did not need tying. He had stumbled across one of those "digesting" cases. Tumor had ruptured and the contents were being extruded into the peritoneal cavity.

> He opened the abdomen and was not a little surprised, after so large an aperture, to find only a glutinous substance stop up this origin; all the difficulty was to remove it; he tried his probes and fingers, but all in vain; it was so slippery that it eluded every touch and the strongest hold he could take of it. No words of mine could excel or improve this description of the extraordinary stuff. He took a strong fir-splinter, about the end of which he wrapped some loose lint, and thrust it into the wound, and by turning and winding it, extracted upwards of two yards in length of a substance thicker than any jelly, or rather like glue that is first made and hung out to dry; its width was upwards of ten inches. This was followed by nine full quarts of such matter as is observed in steatomatous and atheromatous tumors, with several hydatids, of various sizes, containing a yellowish serum, the least of them bigger than an orange, which seemed to be part of the distruded ovarium.

Of course the hydatids were secondary cysts. He closed the wound in the method of his time and (Dr. Greig Smith has not noticed) he drained the peritoneum, "he kept in the lower part of the wound a small tent, which discharged some serosities at every drainage for four or five days."

Let us now suppose that Margaret Millar, of 1701, had fallen into my hands in the year 1897, what difference would there have been in the operation? The anesthetic could have enabled me to empty the abdomen in less time by a stream of warm water, and to empty it more thoroughly, and I should have used a glass drainage tube instead of a tent; and after these differences there is positively nothing. Probably my cleansing of these cases is wholly unnecessary, for after getting quit of the bulk of the gelatinous stuff and thereby reducing the work of the overladen peritoneum, that industrious membrane could nearly always finish the task satisfactorily, as it did in Houston's case. Now we have gone clean daft about modern drainage. We have forgotten all about our father's tents and think drainage a new thing. It is nothing of the sort ; so that Houston's operation was not only an ovariotomy, "a surgical operation by which it was sought to cure a tumor of an ovary," but it was a successful ovariotomy, the first of its kind, a record success. It was done in a fashion almost up to date ; in fact, if I leave out the doubtful washing, I couldn't have done it better myself, and with this little explanation I hope Dr. Greig Smith will remain satisfied.

The horror of the thing remains. For a hundred and ten years the surgical world was blind to the lesson, and it took us another seventy years to struggle into the light of this great fact.

7 THE CRESCENT.

